# Evaluating Endoscopic Retrograde Cholangiopancreatography (ERCP) Outcomes in the Management of Common Bile Duct Stones With a Focus on Difficult Stones: A Retrospective Single-Center Study on Bile Duct Navigation From Kashmir, North India

**DOI:** 10.7759/cureus.86956

**Published:** 2025-06-29

**Authors:** Shaheen Nazir, Waseem A Khan, Zubair Jan, Shahid Sulayman, G M Gulzar, Jaswinder S Sodi

**Affiliations:** 1 Department of Gastroenterology, Sher-i-Kashmir Institute of Medical Sciences, Srinagar, IND

**Keywords:** common bile duct stones (cbds), difficult cbd stone, electrohydraulic (ehl), endoscopic papillary large balloon dilation (eplbd), endoscopic retrograde cholangiopancreatography (ercp), mechanical lithotripsy (ml)

## Abstract

Background: Biliary tract diseases place a significant burden on healthcare in Kashmir. Endoscopic retrograde cholangiopancreatography (ERCP) is the gold standard for managing common bile duct stones (CBDS). However, challenging CBDS present considerable obstacles and complicate patient care, highlighting the necessity for specialized expertise and collaborative approaches. This study evaluates the effectiveness and safety of ERCP in treating CBDS, particularly focusing on difficult-to-manage cases, at a tertiary care center in the Kashmir region of North India.

Objective: This study aimed to investigate the therapeutic outcomes and safety profile of ERCP in CBDS management, emphasizing exploring optimal strategies and modalities for successfully removing complex and difficult-to-treat stones.

Methods: A retrospective study was conducted on ERCPs performed between March 2024 and March 2025 at Sher-i-Kashmir Institute of Medical Sciences (SKIMS) in Srinagar, Kashmir, India. The efficacy of ERCP in clearing CBDS was assessed, and complications were noted. Patients with large (≥1.5 cm) stones of difficult morphology, such as barrel or square-shaped, or those with hard consistency, or those associated with stricture/narrowing seen on prior imaging or cholangiogram were classified as having "difficult" stones. A retrospective review of medical records analyzed patients with CBDS who underwent ERCP, evaluating procedure success, complications, and outcomes, with a focus on effectiveness in difficult CBD.

Results: The study achieved an overall CBDS clearance rate of 650/682 (95.3%), with a clearance rate of 160/190 (84.2%) for difficult stones. For difficult stones, endoscopic papillary large balloon dilation (EPLBD) alone was successful in 117/190 (61.5%) cases. Additional interventions, including mechanical lithotripsy (ML) in 18/190 (9.4%) cases, extracorporeal shock wave lithotripsy (ESWL) in 13/190 (6.8%) cases, and electrohydraulic lithotripsy (EHL) in 12/190 (6.3%) cases, were required. The overall success rates for these interventions were as follows: EPLBD 117/175 (66.8%), ML 18/30 (60%), ESWL 13/20 (65%), and EHL 12/13 (92.3%). Surgical intervention was required in 30/190 (15.7%) cases. Complications occurred in 82 (12%) of overall cases and 13 (6.8%) of difficult stone cases.

Conclusion: In conclusion, our findings underscore the value of ERCP in managing CBDS, particularly challenging ones, with high efficacy and an acceptable safety profile. This supports its continued use as a primary treatment option for complex CBDS. While complications related to ERCP do occur, they are primarily associated with sphincterotomy/precut procedures for gaining access to the CBD. Notably, managing difficult stones does not appear to increase the complication rate, further strengthening ERCP's role in the treatment of complex biliary cases.

## Introduction

The management of common bile duct stones (CBDS) has undergone a paradigm shift with the introduction of endoscopic retrograde cholangiopancreatography (ERCP) [1,2]. ERCP has notably decreased the need for surgical interventions by offering essential therapeutic benefits and precisely identifying bile duct obstructions [2]. However, challenging CBDS present considerable difficulties for endoscopists, with stone clearance often proving elusive in cases involving large stones with difficult morphology, multiple large stones, periampullary diverticula, or previous biliary tract procedures [3,4]. In geriatric patients with multiple comorbidities, the risk of complications increases, making treatment even more daunting [5]. The European Society of Gastrointestinal Endoscopy recommends endoscopic papillary large balloon dilation (EPLBD) as the primary strategy for removing difficult CBDS [6]. Nevertheless, a subset of patients presents with challenging CBDS that defy standard endoscopic approaches, necessitating alternative strategies [4]. While a uniform definition is lacking, difficult CBDS are typically classified into three subtypes based on stone morphology (size >15 mm, irregular shape, and hard consistency), papillary accessibility (anatomical variations), and patient-specific factors (coagulopathy, anticoagulant use, and advanced age >65 years) [4,6,7]. Difficult stones often require additional measures beyond EPLBD, including mechanical lithotripsy (ML), extracorporeal shock wave lithotripsy (ESWL), electrohydraulic lithotripsy (EHL), and laser lithotripsy (LL) [7-10]. Surgery remains an alternative if stone extraction proves unsuccessful or advanced facilities are unavailable [10,11]. Ultimately, treatment decisions should be customized to meet individual patient needs, taking into account the availability of experienced specialists and resources [7,10]. The burden of biliary tract disease is substantial in Kashmir, with a significant incidence of oriental cholangiohepatitis (OCH)-related CBDS [12]. These stones are often characterized by their sturdy and large morphology, frequently associated with strictures, which makes extraction even more challenging [12,13]. This study aims to elucidate the demographic, clinical, and procedural characteristics of patients undergoing ERCP for CBDS in our region. We evaluated the technical success rate and complications associated with ERCP, as well as the impact of stone size and morphology on treatment outcomes. By shedding light on the realities of ERCP practice in our region, this study seeks to bridge the gap between evidence-based guidelines and real-world experience, apprising strategies to optimize CBDS management and improve patient outcomes in resource-limited settings.

## Materials and methods

Study design

This hospital-based retrospective study was conducted in the Department of Gastroenterology at Sher-i-Kashmir Institute of Medical Sciences (SKIMS) in Srinagar, Kashmir, India. Given the retrospective nature of this study, the requirement for ethical clearance was waived by the Institutional Ethics Committee, ensuring compliance with institutional policies and regulations.

Inclusion and exclusion criteria

We retrospectively analyzed data from all ERCP procedures performed at our institution between March 2024 and March 2025. Consecutive patients with CBDS who underwent therapeutic ERCP were included.

Patients with acute suppurative cholangitis, concomitant malignant stricture, IgG4 disease/autoimmune pancreatitis (AIP), pancreatic biliopathy, portal cavernous cholangiopathy, or other indications besides CBDS were excluded. Pregnant patients and those with altered anatomy post-surgery were also excluded.

ERCP procedure

ERCP procedures were performed in the prone position using a standard side-viewing endoscope (Pentax (Tokyo, Japan)/Olympus (Shinjuku City, Tokyo, Japan)) with light sedation (midazolam/propofol). Prophylactic indomethacin CBD suppositories were administered before the procedure. After wire-guided CBD cannulation by papillotome (Ultratome XL/Jagwire Revolution guidewire (Boston Scientific, Marlborough, Massachusetts, United States)), a cholangiogram was obtained to guide further management.

Outcome measures

The primary outcome measure was the complete stone extraction rate, which was considered a successful ERCP. Stone extraction was done by a stone extraction balloon (Boston Scientific) or an Endoline stone extraction basket. Secondary outcome measures included the need for additional interventions, such as EPLBD by controlled radial expansion (CRE) balloon (Boston Scientific), ML by Cook Medical mechanical lithotripter (Bloomington, Indiana, United States), ESWL by Healthy Ware-Dornier compact (Healthware Private Limited, Hyderabad, India), or EHL (Boston Scientific). A decision regarding the need for assistive modalities was taken considering the diameter of the lower end of the CBD, size, number, and shape of the stone.

Large balloon dilation was performed by selectively cannulating the CBD, placing a guidewire, and inserting a large balloon dilator (12-15 mm). The balloon was inflated for 30-90 seconds, then deflated, and removed, followed by stone extraction using a balloon or basket.

ML involved selectively cannulating the CBD, placing a guidewire, and inserting a mechanical lithotriptor (Soehendra, Cook Medical, Bloomington, Indiana, United States). The stone was captured using a basket, and mechanical force was applied to break it. Stone fragments were then removed by balloon or basket sweeps.

As per our hospital protocol, due to the high cost of EHL, large stones not cleared by the above modalities were next subjected to ESWL if found feasible. Nasobiliary tube (NBT) was placed in the same or next session. ESWL was performed by positioning the patient on a fluoroscopy table, following, and localizing the stone using fluoroscopy. Shock waves were then applied to fragment it. A follow-up ERCP was done for stone clearance of the CBD.

EHL with SpyGlass/DS system (Boston Scientific) was performed. The device features a bipolar lithotripsy probe paired with a charger generator and aqueous medium, generating high-frequency electrohydraulic pressure waves that break down bile duct stones. The procedure involved selective cannulation of the CBD, initial cholangiogram, small endoscopic sphincterotomy, and balloon dilation, followed by SpyGlass-guided EHL. Stone fragmentation and removal were then achieved using an extraction balloon and/or basket.

Patients requiring ESWL or EHL, or those with lower-end CBDS in a non-dilated or narrowed duct or with strictures, were treated with plastic stent placement (Boston Scientific). These patients were then scheduled for a follow-up session for stone clearance after 12 weeks.

Stone removal attempts were limited to two unless the patient had a concurrent stricture being treated with a sequential stenting protocol. In such cases, additional attempts were considered. Complications were identified and classified based on established standard definitions.

Difficult stones were defined as stones ≥1.5 cm in size, those with irregular shape like barrel or square, or multiple large CBDS, at least one more than 1.5 cm (≥3). CBD clearance confirmed by balloon-occlusive cholangiogram was considered a successful ERCP. Failed ERCP was defined when a stone couldn't be removed despite assistive stone-crushing modalities, requiring surgery.

Data collection

We collected and analyzed data on the following parameters: patient demographics, stone characteristics (including size, number, and location), biliary anatomy (such as CBD diameter and presence of associated strictures), ERCP and stone clearance modalities used (procedural detail outcome and complications), and follow-up data (including stone clearance rates and complication).

Statistical analysis

Statistical analysis was performed using IBM SPSS Statistics for Windows, Version 20.0 (Released 2019; IBM Corp., Armonk, New York, United States). Continuous variables were expressed as mean and standard deviation (mean (SD) and range). Quantitative data between groups were compared using Student's t-test, the Mann-Whitney U test, and the Kruskal-Wallis test. Pearson's chi-squared test and Fisher's exact test were used for categorical data. Odds ratios were used to determine the strength of associations. All p-values were two-tailed, and a p-value of <0.05 was considered statistically significant.

## Results

A total of 1,338 elective ERCP procedures, performed between March 2023 and March 2024, were included in the study after applying selection criteria. Emergency ERCP procedures, including those for cholangitis where upfront stone extraction was not attempted, were excluded from this study.

The procedures comprised 414 (30.9%) cases of malignant diseases and 924 (69.1%) cases of benign diseases. Choledocholithiasis (CBDS) accounted for the majority (682/924,73.8%) of benign disease procedures. Table 1 summarizes the demographic and biliary parameters of these patients.

**Table 1:** Patient demographics and stone characteristics

Parameter	Value
Mean age (years)	50.4±18.1
Age range (years)	25-85
Male-to-female ratio	1:1.2
Post-cholecystectomy stones	10%
Post-choledochotomy stones	2%

The characteristics of difficult stones in our study are presented in Table 2. Among the patients, 682/924 (73.8%) underwent ERCP for CBDS, 110/924 (11.9%) for pancreatic diseases, and 132/924 (14.2%) for conditions such as hydatid disease with biliary communication, choledochal cyst with stones, biliary ascariasis, portal biliopathy, etc. Among the CBDS, 490/682 (72%) were non-difficult stones, while 190/682 (28%) were difficult stones.

**Table 2:** Characteristics of difficult stones *CBDS: common bile duct stone **Large stone: diameter equal or greater than 1.5 mm

Difficult CBDS*	(n=190; 26.38%)
Square-shaped stones	(n=15; 7.8%)
Distal CBD narrowing	(n=35; 18.4%)
Single large** stones	(n=50; 26.3%)
Multiple large stones	(n=90; 47.3%)

**Figure 1:**
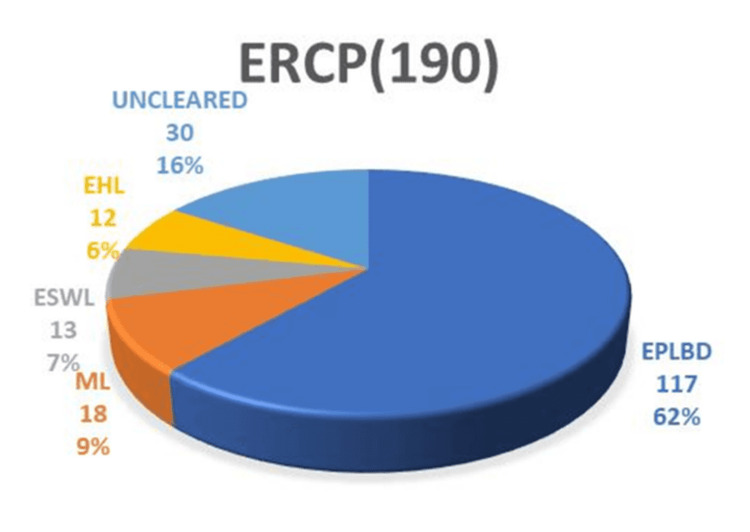
Distribution of modalities used to manage difficult stones shown as n (%) ERCP: endoscopic retrograde cholangiopancreatography; EHL: electrohydraulic lithotripsy; ML: mechanical lithotripsy; EPLBD: endoscopic papillary large balloon dilation; ESWL: extracorporeal shock wave lithotripsy

ERCP was highly effective in clearing the common bile duct (CBD), with an overall success rate of 650/682 (95.3%). All non-difficult stones were successfully removed in a single session using endoscopic sphincterotomy, balloon, and/or basket sweeps, achieving a 490/490 (100%) success rate. Difficult stones, on the other hand, typically require 1.2 sessions for successful removal, necessitating additional interventions following papillotomy. The management of difficult stones in our study cohort involved various modalities. Figure 1 illustrates the distribution of modalities used to manage difficult stones.

**Figure 2:**
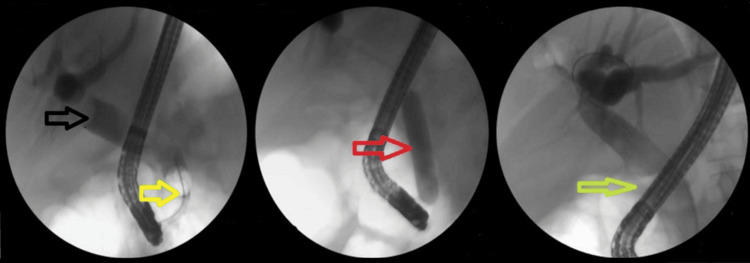
A case where EPLBD was used to manage difficult stones. Black arrow shows the large stone, yellow arrow shows the extraction balloon, red arrow shows the EPLBD, and green arrow shows the occlusive cholangiogram revealing clear CBD EPLBD: endoscopic papillary large balloon dilation; CBD: common bile duct

Notably, the majority of difficult stones were cleared in the first session using EPLBD. The overall success rate of EPLBD with endoscopic sphincterotomy was 607/682 (89%). Only 29/190 (15.2%) of cases of EPLBD procedures were not attempted upfront due to narrow lower ends or strictures (Figure 2). In these cases, stenting was performed first, followed by other modalities in subsequent sessions.

**Figure 3:**
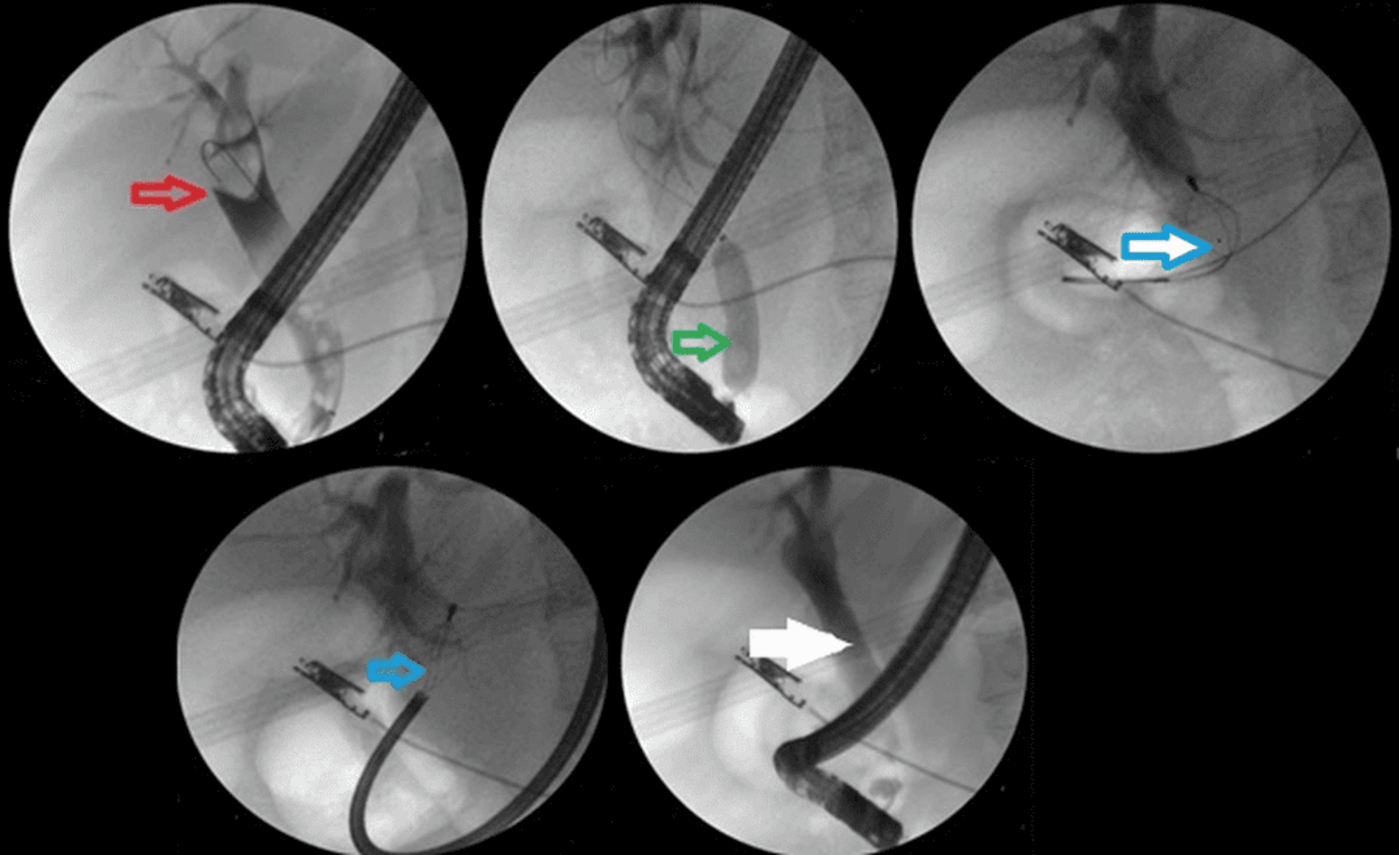
A case where ML was used to manage difficult stones. Red arrow shows the large stone, green arrow shows the EPLBD, blue arrow shows the ML, and white arrow shows the occlusive cholangiogram revealing clear CBD ML: mechanical lithotripsy; EPLBD: endoscopic papillary large balloon dilation; CBD: common bile duct

**Figure 4:**
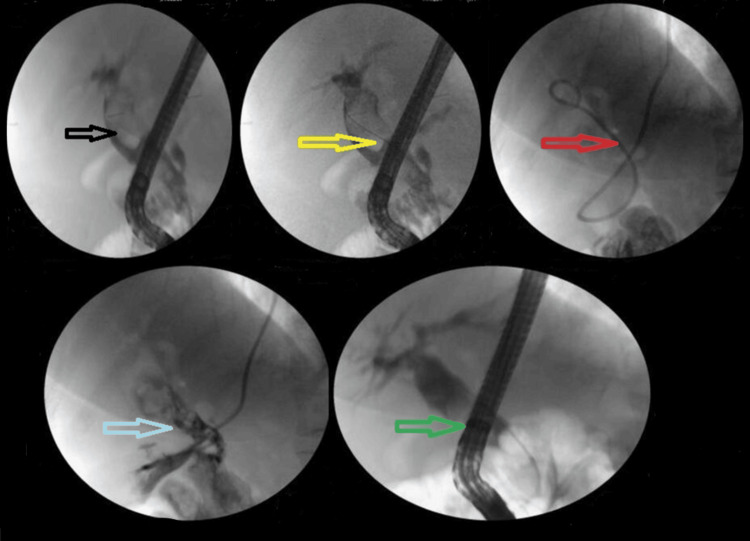
Placement of NBD followed by ESWL. Black arrow shows the large stone, yellow arrow shows the guidewire placed in CBD during ERCP, red arrow shows the NBD, blue arrow shows the pulverized stone after ESWL, and green arrow shows the occlusive cholangiogram revealing clear CBD NBD: nasobiliary drainage; ESWL: extracorporeal shock wave lithotripsy; CBD: common bile duct; ERCP: endoscopic retrograde cholangiopancreatography

**Figure 5:**
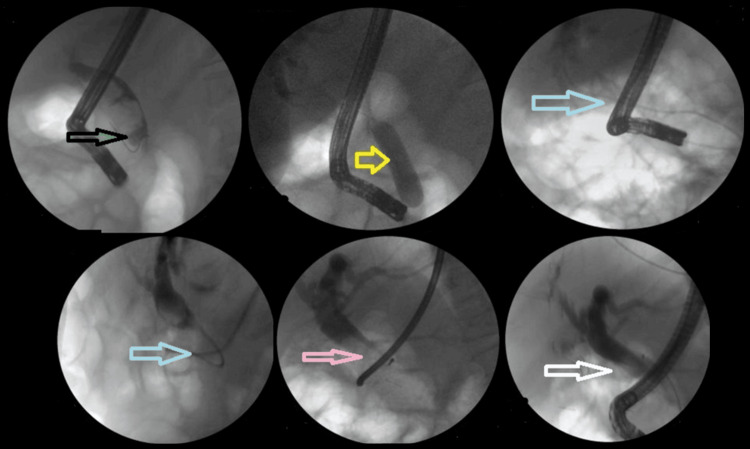
EHL. Black arrow shows the large stone, yellow arrow shows the EPLBD, blue arrow shows the EHL targeting stone, pink arrow shows the pulverized stone, and white arrow shows the occlusive cholangiogram revealing clear CBD EHL: electrohydraulic lithotripsy; EPLBD: endoscopic papillary large balloon dilation; CBD: common bile duct

The addition of ML to EPLBD and endoscopic sphincterotomy resulted in an overall success rate of 625/682 (91.6%). Figure 3 illustrates ML used in a patient. The addition of ESWL (Figure 4) resulted in an overall success rate of 638/682 (93.5%), and the addition of EHL resulted in an overall success rate of 650/682 (95.3%) for the endoscopic removal of stones (Figure 5).

However, in patients with difficult stones, the success rate of ERCP was slightly lower at 160/190 (84.21%). Table 3 shows the success rates of various modalities for difficult stones. Thirty of 190 patients (15.8%) were sent for surgery.

**Table 3:** Follow-up

Stone clearance	Difficult stones	Non-difficult stones
Success rate	84.2%	100%
Number of procedures required	1.2	1

The overall complication rate following the procedure was 82/682 (12%), with 13/190 (6.8%) occurring in difficult stones, accounting for 13/682 (1.9%) of overall cases. Pancreatitis was the most common complication, occurring in 41/682 (6%) of patients. Fortunately, there were no procedure-related deaths. Specific complications related to different modalities are shown in Tables 4-5.

**Table 4:** Success rates of various modalities EPLBD: endoscopic papillary large balloon dilation; ML: mechanical lithotripsy; ESWL: extracorporeal shock wave lithotripsy; EHL: electrohydraulic lithotripsy

Procedure	Success rate (n (%))
Overall	650 (95.3%)
EPLBD	117/175 (66.8%)
ML	18/30 (60%)
ESWL	13/20 (65%)
EHL	12/13 (92.3%)

**Table 5:** Complications of various modalities EPLBD: endoscopic papillary large balloon dilation; ML: mechanical lithotripsy; ESWL: extracorporeal shock wave lithotripsy; EHL: electrohydraulic lithotripsy; CRE: controlled radial expansion

Procedure	Complication (n)
Overall	Perforation (2), bleeding (24), pancreatitis (42), infection (10), other (4)
EPLBD	Perforation (1), bleeding (6), pancreatitis (1)
ML	Broken basket (2), 1 requiring surgical intervention and 1 retrieved by CRE balloon
ESWL	Mild pancreatitis (2)
EHL	Perforation (1), managed conservatively

During a mean follow-up period of 12 months, 69/682 (10%) of patients experienced recurrent CBDS. The majority of these patients underwent repeat ERCP, with a success rate of 98%.

## Discussion

The management of CBDS is paramount, as active stone extraction has been shown to significantly reduce unfavorable outcomes, underscoring its importance even for small stones (<4 mm) [1,6,14]. A retrospective analysis was conducted to critically evaluate the performance of ERCP in the treatment of CBDS, with a focus on complex and refractory cases, at our tertiary care center in Kashmir. Our study provides valuable insights into the real-world efficacy of ERCP in clearing challenging CBDS, bridging the gap between evidence-based guidelines and practical experience, to impart optimized management strategies in resource-limited settings. Our study reveals an overall ERCP success rate of 95.3% in clearing CBDS, aligning with previous research [15,16]. Notably, 27.9% of cases involved difficult stones, which required tailored approaches. EHL proved highly effective, achieving a 92% success rate in clearing challenging stones. Complications occurred in 12% of ERCP procedures, primarily pancreatitis and bleeding, although notably, EHL-related complications were minimal as seen previously [17,18]. The higher complication rate in overall ERCP cases (12%) compared to difficult stone cases (6.8%) can be attributed to the nature of ERCP-related complications. Most complications arise from initial cannulation challenges, such as pancreatitis from inadvertent pancreatic duct cannulation or perforation with precut techniques. In difficult stone cases, where sphincterotomy has already been performed, the cannulation-related issues are minimized, resulting in fewer complications. This suggests that the complexity of cannulation, rather than stone extraction itself, is a significant contributor to ERCP-related complications.

Conventional ERCP methods often fall short in clearing difficult biliary stones in a single procedure. Previous studies report a clearance rate of only 65-70% with EPLBD [19,20]. Our approach, incorporating prolonged balloon dilation (90 seconds), achieved a success of 67% in difficult stones. Our complication profile was similar to previous reports [20,21]. Literature demonstrates the superiority of endoscopic sphincterotomy with large balloon dilation (ES-LBD) in clearing large CBDS (≥13 mm), with a clearance rate of 96.1% versus 74% with conventional sphincterotomy (p<0.001) [2]. ES-LBD also reduces the need for ML, associated costs, and procedure time while maintaining a similar safety profile [16,20,21]. Our findings support the potential of ES-LBD as a preferred approach for managing large CBDS. None of our patients underwent EPLBD without prior sphincterotomy. EPLBD without prior sphincterotomy is associated with higher pancreatitis risk and poorer stone clearance, except for small stones (<8 mm), but may be considered in specific cases with coagulopathy, anatomical challenges, or increased sphincterotomy risks [21]. In our study, we employed endoscopic sphincterotomy before EPLBD, which may have contributed to the lower pancreatitis rate observed. Conversely, the bleeding rate was slightly higher, although most cases were managed conservatively.

While EPLBD is effective for three-quarters of large CBDS, certain complex cases still require lithotripsy. These include large stones, stones lodged in narrow ducts, and intrahepatic stones. Due to the prohibitive cost of peroral cholangioscopy-assisted lithotripsy, ML is a practical alternative in resource-constrained settings [4]. ML utilizes a mechanical shearing force to fragment stones, leveraging a specialized apparatus comprising a basket, traction wire, and metal sheath. Two distinct systems are available: an extra-duodenoscope setup, employed at our institution for emergencies and retrieving entrapped baskets, and an integrated through-the-scope configuration, not available at our center [22-24]. ML is typically reserved for challenging stones, with a success rate of 79-94% and a complication rate of 3.3-17.6% [25-27]. Effective patient selection is paramount, with optimal candidates presenting with sizable (>1.5 cm) and hardened stones inducing substantial biliary obstruction. The existing data underscores ML's impressive efficacy (90-95%) and favorable safety profile [4,22-24]. Our institution's experience corroborates these outcomes in managing recalcitrant CBDS.

ESWL is a valuable adjunctive treatment for difficult CBDS [25,26]. Our study focused on patients with large (>2 cm) and hard stones resistant to endoscopic extraction and ML. Consistent with existing literature [9], our results show ESWL to be highly effective, with an 88% stone clearance rate and no major complications.

ESWL uses electrohydraulic energy to break up bile duct stones under fluoroscopy guidance [9]. The procedure has a stone clearance rate of 84.4-90.2% [9,25,26]. However, it has limitations, including the need for an NBT or a T-tube, multiple sessions, and anesthesia [9,25]. Complications occur in 9.1-15.9% of cases, including hemobilia and cardiac arrhythmia [9,25,26]. The European Society of Gastrointestinal Endoscopy recommends ESWL when conventional lithotripsy fails and cholangioscopy-guided lithotripsy is not available [6].

For patients with refractory CBDS, single-operator cholangioscopy (SOC)-guided intraductal lithotripsy using EHL or LL achieves high stone clearance rates (73-97%). EHL and LL fragment stones through shock waves. EHL uses a high-voltage spark to generate a shock wave, while LL employs pulsed laser energy to create thermal expansion and fragmentation [10,17]. Both techniques require direct visualization for safe and precise stone targeting. These are minimally invasive, promising alternatives to laparoscopic common bile duct exploration (LCBDE) [10,17]. While generally safe, cholangioscopy carries a risk of cholangitis, emphasizing the need for prophylactic antibiotics. Comparative analysis of endoscopic and surgical approaches revealed conflicting evidence, emphasizing the need for individualized treatment strategies. Studies show that SpyGlass-guided lithotripsy and LCBDE have comparable morbidity and mortality [10].

Our use of EHL for large CBDS aligns with existing evidence, demonstrating its effectiveness in clearing difficult stones. EHL offers a high success rate (92%) and low complication rate (3.8%), making it a valuable option for avoiding surgery [18]. Advanced endoscopic techniques, like SpyGlass-guided LL and EHL, have been shown to achieve comparable stone clearance rates to LCBDE, highlighting the efficacy of endoscopy in managing complex CBDS with minimal additional training required for endoscopists proficient in ERCP, unlike surgeons who require longer learning curve training.

Overall, our study demonstrates the efficacy and safety of ERCP in clearing CBDS, with a success rate comparable to international standards. The use of EHL significantly enhances the clearance rate of difficult CBDS. Cholangioscopy-assisted EHL, in particular, allows for the precise visualization and targeting of stones, minimizing the need for invasive surgery. Our department's experience with EHL has been promising, with a high clearance rate and minimal referrals for surgery.

A well-performed papillotomy followed by balloon dilatation can clear stones in most cases, minimizing complications and the need for surgical interventions. ERCP is a cost-effective, minimally invasive alternative to surgery, with shorter recovery times, making it suitable for patients with comorbidities or limited access to advanced surgical care. In our clinical context, where OCH contributes to recurrent CBDS, ERCP offers a more prudent and efficacious approach than repetitive surgical interventions. Our findings suggest that endoscopic management should remain the initial treatment option for difficult CBDS, reserving surgery for cases where stone extraction fails or advanced facilities are unavailable.

Limitations

Our study has several limitations, including its retrospective design and the relatively small sample size over one year. Additionally, our study only included patients who underwent ERCP at a single center, which may limit the generalizability of our findings.

Future directions

Future studies should aim to validate our findings in larger, prospective cohorts and explore the long-term outcomes of ERCP in patients with difficult CBDS.

## Conclusions

Our study unequivocally demonstrates the exceptional efficacy of ERCP in managing CBDS, including complex cases, within a single-center setting in Kashmir, North India. Our findings robustly support the adoption of ERCP as a first-line treatment modality for patients with challenging CBDS, emphasizing the paramount importance of meticulous patient selection and pre-procedural evaluation in determining the success of ERCP. This study reaffirms the value of ERCP, particularly in regions with limited access to advanced surgical facilities, and highlights the critical role of operator expertise, resource availability, and personalized treatment strategies in optimizing outcomes.
